# Surgeon-oriented three-dimensional planning for supracondylar humeral malunion

**DOI:** 10.1016/j.xrrt.2026.100801

**Published:** 2026-06-18

**Authors:** Hidemasa Yoneda, Hirotaka Sugiura, Masaomi Saeki, Nobunori Takahashi, Michiro Yamamoto

**Affiliations:** a Department of Human Enhancement and Hand Surgery, Nagoya University Graduate School of Medicine, Showa-ku, Nagoya, Japan; b Department of Orthopedics, Aichi Medical University, Nagakute, Japan

**Keywords:** Supracondylar fracture, Malunion, Three-dimensional osteotomy, Osteotomy planning, Landmark invariance, Computer-aided surgery

## Abstract

**Background:**

Malunion of supracondylar humeral fractures is often associated with complex three-dimensional (3D) rotational deformities involving the coronal, sagittal, and transverse planes. Pre-operative planning for corrective osteotomy typically requires computer-aided design engineers, limiting surgeons' ability to independently understand deformity patterns and adjust surgical strategies intraoperatively. A practical, surgeon-oriented method for quantifying 3D rotational deformities is, therefore, needed. Herein, we established a surgeon-oriented computational framework that enables quantitative assessment of 3D deformities based on computed tomography–derived 3D bone models and allows surgeons to independently perform pre-operative planning using 3D-computer-aided design software.

**Methods:**

We developed a 3D rotation analysis method based on mirrored bone models of the unaffected humerus. After superimposition of proximal segments, object-specific coordinate systems were defined using a centroid and 2 anatomical landmarks. Rotational deformities were calculated using 3 approaches: wedge osteotomy-based rotation (Method A), 3D rotational osteotomy (Method B), and coronal-plane-only rotation (Method C). Validation was performed using computed tomography data from 5 patients with extra-articular supracondylar humeral malunion. Robustness was assessed by calculating rotational deformities for all 21 landmark-pair combinations derived from 7 predefined landmarks and by evaluating sensitivity to coordinate rounding.

**Results:**

Across all patients, rotational components calculated using Methods A and B showed minimal variability across landmark-pair selections, with median within-patient ranges below 0.02° for all components. Method C demonstrated a larger absolute range but remained consistent relative to the magnitude of rotation. Rounding landmark coordinates to 1 decimal place resulted in only minor changes in calculated rotations, whereas integer-level rounding produced substantial deviations, particularly in transverse-plane components. In addition, the coronal-plane rotational components derived from Methods A and B closely matched the single-axis rotation obtained by Method C, with absolute differences within 3° across all patients.

**Conclusion:**

This study demonstrates a robust and surgeon-oriented method for quantifying 3D rotational deformities in extra-articular supracondylar humeral malunion. The method is insensitive to landmark selection and clinically realistic coordinate precision, which may reduce dependence on dedicated engineering support or outsourced services. Furthermore, consistency between 3D and simplified coronal-plane correction estimates supports the practical applicability of this approach in routine surgical decision-making.

Malunion after supracondylar humeral fractures is uncommon. However, when it occurs, it can lead to significant complications, including esthetic issues, delayed ulnar nerve palsy, limited elbow range of motion, and joint instability.^1^^,^^4^^,^^9^ Therefore, malunions with significant deformities are indicated for surgical osteotomy. Such malunions often involve complex rotational deformities across the coronal, sagittal, and transverse planes, making accurate correction technically demanding. To address these multiplanar deformities, three-dimensional (3D) osteotomy techniques have been developed to anatomically correct alignment across all planes.^10^

Owing to the difficulty of pre-operative planning for 3D osteotomy, recent advancements have incorporated computer simulations to enhance precision.^8^^,^^10^ These systems use 3D computer-aided design (3D-CAD) software to superimpose a mirrored 3D model of the unaffected side onto the affected side, enabling osteotomy planning based on their geometric differences.^10^ In addition, patient-matched instruments (PMIs), created using 3D printing technology, facilitate accurate intraoperative reproduction of the planned correction. By following a guided workflow, surgeons can perform these procedures without explicitly engaging in the underlying mathematical calculations of deformity and correction.

However, in some clinical situations, surgeons may wish to selectively apply only certain components of the simulation results. For example, in children younger than 10 years, sagittal plane remodeling may occur after fracture union, making coronal deformity correction alone a reasonable strategy.^6^ Similarly, extensive correction of axial rotation can reduce the bone contact area at the osteotomy site, leading some surgeons to intentionally limit correction in the transverse plane because of concerns regarding bone healing and clinical outcomes.^8^ In current workflows, such surgeon-driven modifications generally require reanalysis by engineers, thereby increasing preparation time and reducing flexibility.

Although computer-assisted 3D osteotomy techniques enable the precise execution of a predefined plan, they inherently limit the surgeon's ability to independently quantify deformity and adjust the correction based on clinical judgment. To our knowledge, no method has been reported that allows surgeons themselves to quantitatively measure 3D deformities due to malunion and independently generate a pre-operative correction plan using computer assistance. This limitation may stem from the difficulty of decomposing 3D rotational deformities into clinically interpretable components and from the unintuitive nature of 3D rotation. Moreover, measurements based on plain radiographs—while widely accessible—are known to be less accurate than those derived from 3D models reconstructed from computed tomography (CT) data.^7^

Therefore, we established a surgeon-oriented computational framework that enables quantitative assessment of 3D deformities based on CT-derived 3D bone models and allows surgeons to independently perform pre-operative planning using 3D-CAD software (Fig. 1). This framework defines patient-specific coordinate systems for precorrection and postcorrection models and calculates rotational deformities from their relative transformations. In this study, we present the theoretical framework, calculation methods applicable to dome and wedge osteotomies, and executable scripts for surgeon-driven 3D osteotomy planning. We further validated the robustness and numerical stability of these calculations using CT data from multiple patients.

**Figure 1:**
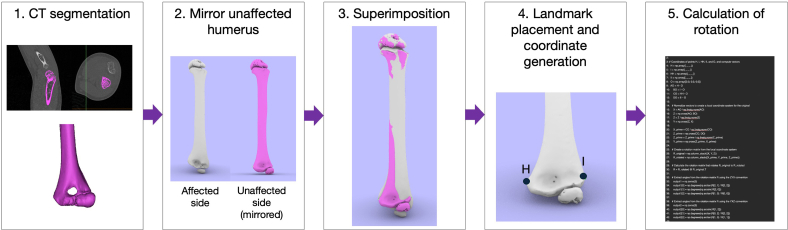
Workflow for surgeon-oriented three-dimensional osteotomy planning. *CT*, computed tomography.

## Materials and methods

### Study design

The proposed framework enables surgeons to calculate rotational deformities using only 2 user-selected anatomical landmarks in combination with a reference centroid, facilitating practical clinical use. Validation was performed using CT data from 5 cases of malunited supracondylar fractures of the humerus.

### Three-dimensional bone model construction

The study was conducted in accordance with the Declaration of Helsinki and approved by the institutional review board. CT scanning and segmentation were performed following a previously reported method.^11^ Bilateral humeri were scanned using a CT scanner (Aquilion ONE 64, Canon Medical Systems, Otawara, Japan; slice thickness 0.5 mm). Bone segmentation was performed using Mimics 21.0 (Materialise, Leuven, Belgium). The resulting 3D surface models were exported in Stereolithography format and imported into Rhino 8.15 (Robert McNeel & Associates, Seattle, WA, USA). The unaffected humerus was mirrored and used as the anatomical reference model.

**Figure 2:**
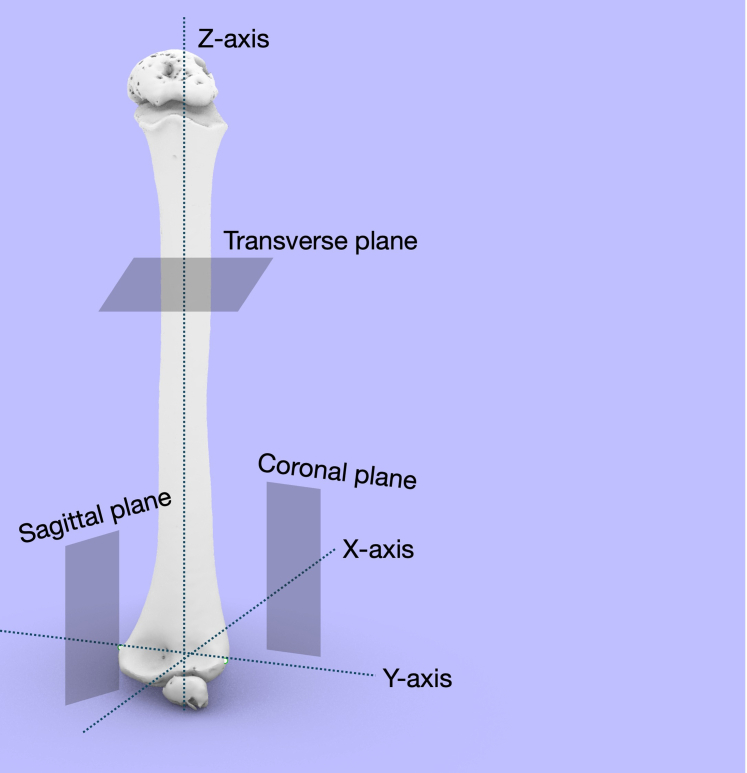
Definition of global axes.

A global coordinate system was defined on the mirrored, unaffected model (Fig. 2). The global Z-axis corresponded to the long axis of inertia of the humerus. The Y-axis was defined by the line connecting the most prominent points of the medial and lateral epicondyles on the transverse plane, and the X-axis was defined as orthogonal to both the Y- and Z-axes.

### Operational workflow for two-point-based rotation measurement

**Figure 3:**
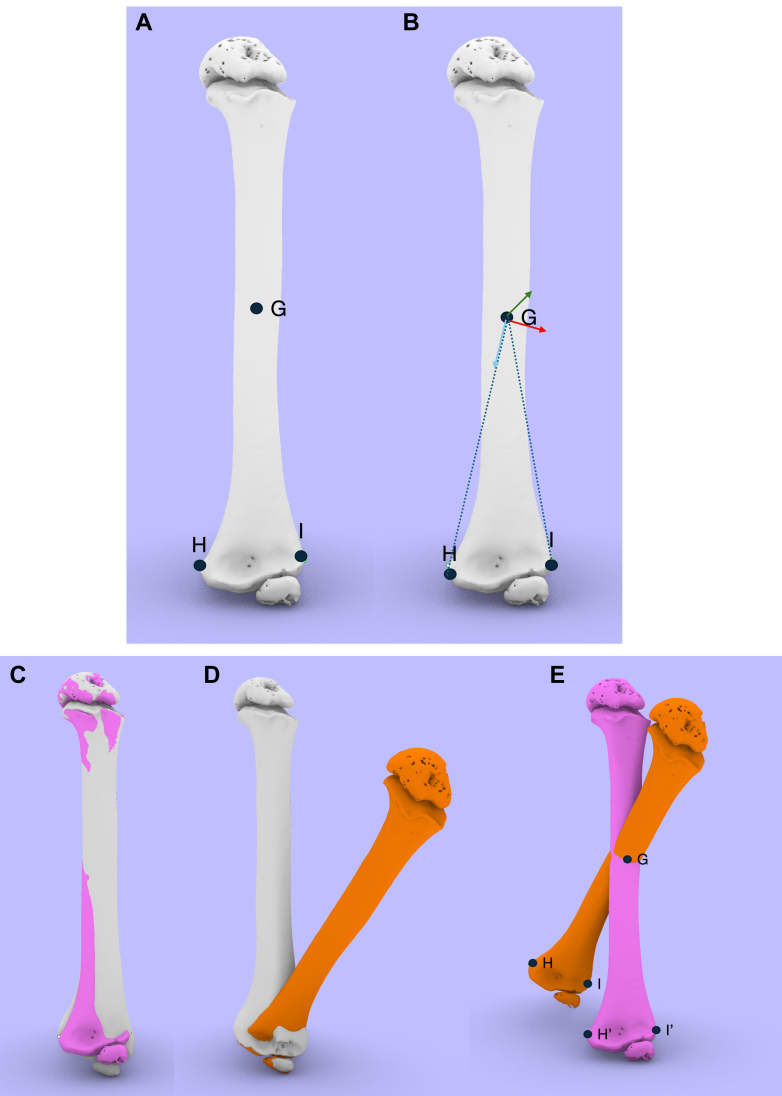
Determination of intrinsic coordinate axes on the humerus model. Two points (*H* and *I*) distal to the malunion and the center of gravity *G* of the object are identified (**A**). The intrinsic coordinate axes of the humerus model are determined using the method shown in Fig. 1 (**B**). The affected side (*white*) and the mirrored healthy side model (points *H*, *I*, and *G*, and *object O*_*1*_, *shown in pink*) are aligned at the humeral head (**C**). A copy of the mirrored healthy side model (points *H′*, *I′*, '*G′*, and *object O*_*2*,_*shown in orange*) is aligned distally at the humerus (**D**). The centroids *G* and *G′* of the 2 mirrored healthy side models are translated to the origin and superimposed (**E**).

In the proposed method, rotational deformities are quantified using only 2 anatomical landmarks placed on the humerus. After constructing the 3D bone models, the centroid of the unaffected humerus was calculated (Fig. 3). Two arbitrary landmarks were then placed on the unaffected model. In clinical practice, these landmarks are typically positioned distal to the malunion, such as on the medial and lateral epicondyles, to enhance reproducibility.

The affected humerus was superimposed onto the unaffected reference model by aligning the proximal segment near the malunion as closely as possible. Given that the humeral head is minimally affected by epiphyseal deformity, it served as a reliable region for alignment. The unaffected model, together with the centroid and the 2 landmarks, was then copied and translated to match the distal segment of the affected humerus while preserving all relative positional relationships.

After superimposition, the centroids of the precorrection and postcorrection models were shifted to coincide at the origin. The coordinates of the centroid and the 2 landmarks before and after correction were recorded and used to construct object-specific coordinate systems.

### Coordinate system definition and rotation calculation

Using the centroid and 2 landmarks, a unique object coordinate system was defined for each bone model. The local X-axis was defined as the normalized vector from the centroid to the first landmark. The local Z-axis was defined as the normalized cross-product of vectors formed by the 2 landmarks relative to the centroid, and the local Y-axis was defined as orthogonal to both X and Z.

**Figure 4:**
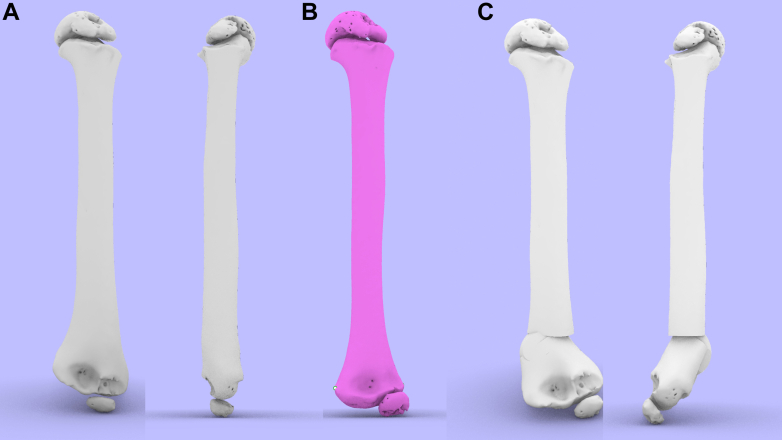
Simulation of dome-shaped osteotomy. Pre-operative images of the affected side (**A**) and the mirrored healthy side (**B**). The simulation of osteotomy using calculated parameters confirms proper reduction (**C**).

**Figure 5:**
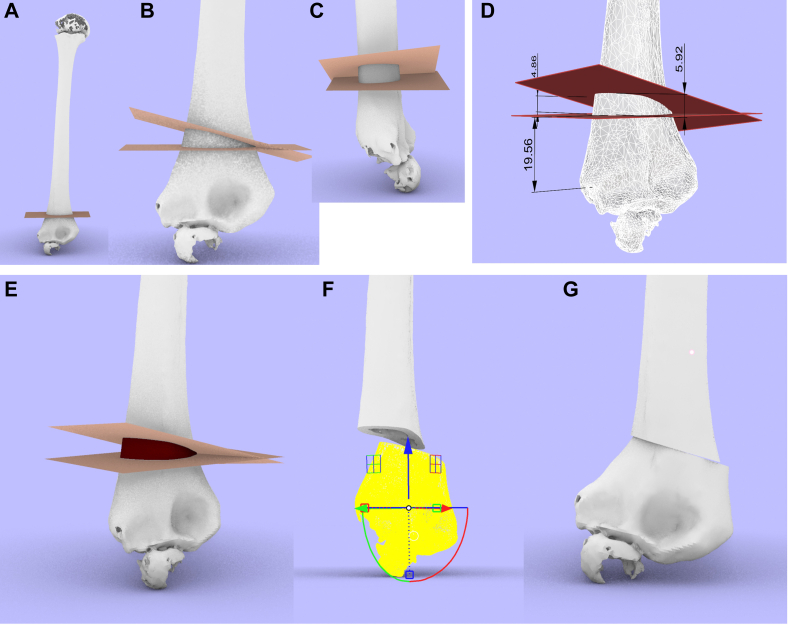
Overview of the closed wedge osteotomy procedure. A plane perpendicular to the bone inertia axis is created proximally to the olecranon fossa and set as the distal osteotomy plane (**A**). This plane is duplicated and rotated around the global X-axis (**B**). The proximal osteotomy plane is then rotated around the global Y-axis to complete the closed wedge osteotomy planes (**C**). The area surrounded by the 2 planes is the area to be resected in a closed wedge osteotomy. Using the measurement function of the computer-aided design (CAD) software, the height of the lateral flexion and lateral extension of this area and the distance from the most prominent part of the epiphyseal nucleus of the lesser head are measured (**D**). By resecting this area and bringing the osteotomy surfaces in contact, the osteotomies in the coronal and sagittal planes are completed (**E and F**). Finally, rotational deformity is corrected by rotating the proximal osteotomy plane around the local Z-axis (**G**).

**Figure 6:**
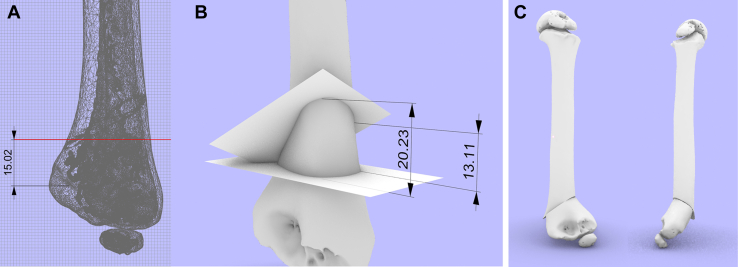
Design of osteotomy when the distal osteotomy plane is set at 15 mm from the medial epicondyle (**A** and **B**) and the result after osteotomy and reduction (**C**).

Rotational deformities were calculated as the relative transformation between the coordinate systems of the affected humerus and the mirrored unaffected reference model. The following 3 calculation methods were evaluated: Method A (wedge osteotomy, Fig. 4), Method B (3D rotational osteotomy, Fig. 5), and Method C (coronal-plane-only rotation, Fig. 6).

#### Rotation calculation methods

Rotational deformities were quantified as the relative transformation between the coordinate systems of the affected humerus and the mirrored unaffected reference model. Three calculation methods were evaluated.

#### Method A: wedge osteotomy-based rotation

It calculates 3 rotational components corresponding to coronal, sagittal, and transverse planes based on the wedge osteotomy theory. This method assumes sequential rotations around orthogonal axes.

#### Method B: three-dimensional rotational osteotomy

It calculates 3D rotational deformities without restricting the order of rotations, corresponding to dome or rotational osteotomy. This method represents the most general form of 3D correction.

#### Method C: coronal-plane-only rotation

It calculates a single rotation angle around the coronal plane (X-axis). This method was designed to simulate clinical scenarios in which correction is intentionally limited to a single plane, such as in younger pediatric patients.

Detailed mathematical formulations are provided in the Supplementary Appendix.

### Validation protocol

#### Validation of invariance to landmark placement

Although the proposed method requires only 2 landmarks for clinical use, validation was intentionally performed using 7 different anatomical landmarks to assess robustness against landmark placement and distance from the centroid. Seven landmarks were selected at reproducible anatomical locations, including the lateral epicondyle, capitulum, medial epicondyle, trochlea, anterior greater tubercle, posterior greater tubercle, and humeral head.

All possible combinations of 2 landmarks were generated, yielding 21 unique point pairs per patient. Rotational deformities were calculated independently for each pair. Variability attributable to landmark selection was assessed using the range and maximum absolute deviation of rotation angles across the 21 combinations.

#### Sensitivity to coordinate rounding

To evaluate numerical robustness to coordinate precision, landmark coordinates were rounded to different decimal levels (3 decimal places [reference], 2 decimal places, 1 decimal place, and integer values), and the entire analysis pipeline was repeated for each condition. Absolute differences from the reference calculation were computed for each rotational component.

#### Outcome measures and implementation

Robustness was quantified using the maximum absolute difference and the 95th percentile absolute difference across landmark combinations. Metrics were summarized on a per-patient basis and across patients. All analyses were performed using Python (version 3.11.11) with NumPy (version 2.0.2; Table I).

**Table I:** Software and programs used for three-dimensional osteotomy planning.

Step	Software	Purpose
Segmentation	Mimics	STL generation
Three-dimensional model alignment	Rhino	Model superimposition
Calculation of rotation	Python/NumPy	Rotation analysis

*STL*, Stereolithography.

## Results

### Invariance to landmark-pair selection

Rotational deformities were computed for each patient using all 21 landmark-pair combinations generated from 7 predefined anatomical landmarks. Method A yielded 3 rotational components (Ax, Ay, Az) corresponding to rotations in the coronal, sagittal, and transverse planes, respectively. Method B yielded 3 components (Bx, By, Bz) using an alternative 3D rotation representation. Method C yielded a single scalar rotation (C), representing rotation around the local X-axis.

Across the 5 patients, the median within-patient range (max–min across the 21 landmark-pair combinations) was 0.004° for Ax, 0.002° for Ay, and 0.012° for Az in Method A. For Method B, the median within-patient range was 0.002° for Bx, 0.001° for By, and 0.012° for Bz. These values indicate that rotational components calculated using methods A and B varied by less than 0.02° across different landmark-pair selections in all planes.

**Figure 7:**
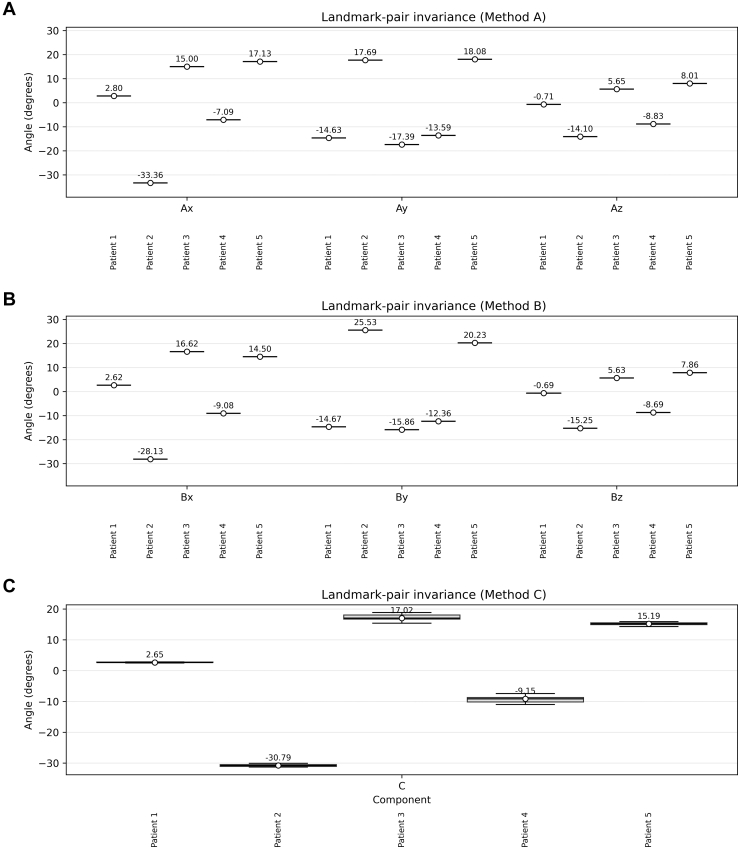
Landmark-pair invariance of rotational measurements calculated using Methods A, B, and C. (**A**) Method A yielded 3 rotational components (Ax, Ay, Az) corresponding to the coronal, sagittal, and transverse planes. (**B**) Method B yielded 3 rotational components (Bx, By, Bz) based on a three-dimensional rotation formulation. (**C**) Method C yielded a single scalar rotation (**C**) around the local X-axis. For each patient, rotational values were calculated using all 21 landmark-pair combinations. Boxplots show the distributions across landmark selections, with open circles indicating median values. In Methods A and B, the boxes appear compressed because variability across landmark pairs was extremely small, indicating high invariance. Panels A and B share a common y-axis scale, whereas panel C is shown on an independent scale. The median coronal-plane rotations (Ax and Bx) were comparable to the single-axis rotation obtained by Method C across patients, with absolute differences within 3°.

For Method C, the median within-patient range across patients was 1.63°. Although the absolute range was larger than that observed in Methods A and B, the variation remained limited relative to the magnitude of rotation measured by this single-axis method. Patient-level ranges and summary statistics are provided in Table II, and distributions across patients are shown in Figure 7.

**Table II:** Within-patient range of rotational measurements across landmark-pair combinations.

Component	Median range (°)	Min–max across patients (°)
Method A		
Ax	0.00404	0.00150-0.01110
Ay	0.00185	0.000848-0.00997
Az	0.01179	0.00427-0.03253
Method B		
Bx	0.00171	0.000744-0.00347
By	0.00144	0.000962-0.00243
Bz	0.01154	0.00426-0.03151
Method C		
Cx	1.63293	0.38317-3.58815

### Sensitivity to coordinate rounding

Rounding landmark coordinates to 2 decimal places resulted in minimal numerical differences relative to the reference calculation performed using 3 decimal places. Across patients, the median maximum absolute difference (max|Δ|) was 0.034° for Ax, 0.020° for Ay, and 0.084° for Az in Method A and 0.011° for Bx, 0.0074° for By, and 0.090° for Bz in Method B. For Method C, the median max|Δ| was 0.0036°, indicating minimal sensitivity to this level of rounding.

When coordinates were rounded to 1 decimal place, the median max|Δ| remained below 1° for all rotational components in Methods A and B, with median values of 0.288° for Ax, 0.215° for Ay, and 0.778° for Az and 0.088° for Bx, 0.089° for By, and 0.769° for Bz. For Method C, the median max|Δ| was 0.0358°. These results show that rounding to 1 decimal place introduced only small changes relative to the calculated correction angles.

With integer rounding, larger deviations were observed, particularly in the transverse components Az and Bz. Median max|Δ| values exceeded 9° for these components, and maximum values exceeded 30°, indicating substantial sensitivity at this level of coordinate precision. Table III provides detailed summary statistics for all rounding conditions.

**Table III:** Sensitivity of rotational measurements to landmark coordinate rounding.

Component	2 decimals median max|Δ| (95th percentile) (°)	1 decimal median max|Δ| (95th percentile) (°)	Integer median max|Δ| (°)
Method A			
Ax	0.034 (0.046)	0.289 (0.333)	2.52
Ay	0.020 (0.061)	0.215 (0.296)	1.87
Az	0.084 (0.123)	0.778 (0.901)	9.33
Method B			
Bx	0.011 (0.016)	0.088 (0.113)	2.31
By	0.007 (0.024)	0.089 (0.136)	1.94
Bz	0.090 (0.122)	0.769 (0.887)	9.18
Method C			
Cx	0.004 (0.004)	0.036 (0.043)	0.42

In addition, across all patients, the coronal-plane rotational components obtained by Methods A (Ax) and B (Bx) closely matched the single-axis rotation calculated by Method C, with absolute differences consistently within 3°. This finding indicates that the magnitude of coronal-plane correction was comparable between the 3D correction models and the simplified single-plane model.

## Discussion

Herein, we introduced a surgeon-oriented method for quantifying 3D rotational deformities associated with extra-articular supracondylar humeral malunion. The proposed framework is based on mirroring the unaffected humerus and superimposing it onto the affected side, followed by calculating the relative rotational discrepancy between the corresponding coordinate systems. By defining object-specific coordinate axes for each 3D model, the rotational difference directly represents the correction required to restore the target alignment of the distal humeral joint. Once the surgeon determines the osteotomy planes and the sequence of correction, the necessary correctional values can be extracted and applied intraoperatively based on clinical judgment.

A key finding of this study is that the calculated rotational corrections were highly robust to landmark selection. Although only 2 anatomical landmarks are required for routine clinical application, systematic validation using all 21 possible combinations of 7 predefined landmarks demonstrated extremely small variability in the calculated rotation angles. This finding indicates that the proposed method does not rely on precise or rigidly defined landmark placement and is, therefore, less sensitive to surgeon-dependent landmark selection. Such robustness is particularly advantageous in clinical settings, where minor variability in landmark placement is often unavoidable.

In addition, sensitivity analysis demonstrated that the method is numerically robust to clinically realistic coordinate precision. Rounding landmark coordinates to 1 decimal place resulted in only minimal changes in calculated rotation angles, whereas integer-level rounding led to substantial variability in certain rotational components. These results suggest that the proposed approach can be reliably applied using standard 3D imaging and modeling software without requiring submillimeter coordinate accuracy, which is often difficult to achieve in routine clinical practice.

An additional clinically relevant observation was that the magnitude of coronal-plane rotation derived from the 3D correction models (Ax in Method A and Bx in Method B) closely matched the single-axis rotation calculated by Method C in all patients. The absolute differences between these values remained within a small range. This finding suggests that, even in the presence of complex 3D deformities, the correction required around the coronal plane can be reliably estimated using a simplified single-plane osteotomy model. Given that coronal-plane deformity is often the primary determinant of functional and cosmetic outcome in supracondylar humeral malunion,^6^ this result supports the practical utility of simplified correction strategies when the primary surgical objective is coronal alignment.

The rotation concept applied in this study represents the orientation of a rigid body or reference coordinate system in 3D space. By defining the coronal, sagittal, and transverse planes of the humerus as the reference coordinate system, the calculated rotational values can be interpreted intuitively and directly translated into surgical correction. Considering that Euler angle-based rotations are sequence-dependent, prioritization of the correction sequence is required. In clinical practice, coronal-plane deformities, such as varus or valgus, are typically corrected first because of their greater impact on complications and appearance, followed by sagittal-plane deformities affecting flexion–extension, and finally transverse-plane deformities, which generally have less influence on clinical outcomes. This sequence-dependent framework aligns well with conventional surgical decision-making and allows selective correction of individual deformity components.

Several computer-assisted osteotomy techniques using PMIs have demonstrated high accuracy, often within 1° and 1 mm of simulated values.^2^^,^^3^^,^^5^ However, these systems require additional time, cost, and engineering support, with PMI fabrication typically taking 1 to two months and incurring substantial additional expense.^2^ Furthermore, if discrepancies arise between simulation results and the surgeon's expectations, further consultation and preparation may be necessary. In contrast, the present method enables surgeons to independently perform pre-operative planning and calculate correction angles without relying on engineers or outsourced services. With sufficient familiarity, the entire workflow, from 3D model construction to rotation analysis, can be completed within approximately 1 h, potentially reducing both cost and preparation time.

This study has some limitations. First, because the rotation matrix is sequence-dependent, inappropriate prioritization of the correction order may result in different postosteotomy alignment. Second, bilateral CT imaging increases radiation exposure and may increase medical costs, although this can be mitigated through optimized scanning protocols. Because the costs of CT imaging, software licenses, and PMI fabrication vary substantially among countries and institutions, formal cost analysis was beyond the scope of the present study. Third, accurate reproduction of the calculated correction during surgery is required, and achieving precise osteotomy execution remains technically demanding. In this regard, PMI-based techniques may still offer advantages when exact replication of simulation results is the primary objective. Furthermore, the present study validated the computational robustness and numerical stability of the proposed framework; however, post-operative correction accuracy in actual surgical cases was not evaluated. Thus, future studies comparing planned and achieved correction accuracy are warranted. In addition, the proposed method is limited to extra-articular deformities and is not applicable to intra-articular fractures. Finally, implementation requires CAD software, which may be costly, and surgeons must ensure that any software used complies with local regulatory requirements for medical applications.

## Conclusion

This study established a practical and robust method for quantifying 3D rotational deformities in extra-articular supracondylar humeral malunion using 3D bone models. By demonstrating robustness to landmark selection and coordinate precision, and by showing consistency between 3D and simplified coronal-plane correction estimates, the proposed approach supports surgeon-driven pre-operative planning without dependence on patient-matched instrumentation or engineering assistance.

## Disclaimers:

Funding: This work was supported by 10.13039/501100001691Japan Society for the Promotion of Science (JSPS) 10.13039/501100001691KAKENHI (grant numbers 21H03288 and 24K12131).

Conflicts of interest: The authors, their immediate family, and any research foundation with which they are affiliated have not received any financial payments or other benefits from any commercial entity related to the subject of this article.
