# Supplementary material for: Surgeon-oriented three-dimensional planning for supracondylar humeral malunion
Source: JSES Rev Rep Tech. 2026 Jun 18;6(4):100801. doi: 10.1016/j.xrrt.2026.100801 (PMC13400223; doi:10.1016/j.xrrt.2026.100801)
Supplement: Appendix [file mmc1.docx]

**Appendix A. Mathematical basis of rotation calculation**

**1. Mathematical basis for rotation transformations**

*1.1. Rodrigues rotation formula and rotation matrices*

According to the laws governing rotation and vectors, when a rotation matrix R is applied to vector a, the resulting vector c is given by

$$\begin{aligned} c= R\cdot a\#\left( 1 \right) \end{aligned}$$

When the type of rotation is defined, the rotation matrix representing this rotation can be calculated using Rodrigues’ rotation formula. It allows us to calculate the rotated vector **v′**of an original vector **v** after rotating it by an angle θ around a rotation axis represented by the unit vector **k**.

$$\begin{aligned} v^{'}=v\cos\left( \theta\right)+\left( k\times v \right)\sin\left( \theta\right)+k\left( k\cdot v \right)\left( 1-\cos\left( \theta\right) \right)\#\left( 2 \right) \end{aligned}$$

Denoting the rotation matrix by R_ro_(θ), we can express this relationship as:

$$\begin{aligned} v^{'}=R_{ro}\left( \theta\right)\cdot v\#\left( 3 \right) \end{aligned}$$

To compute R_ro_(θ), substitute **v**= (v1, v2, v3) and k= (k1, k2, k3) into Equation (2), leading to the following three equations:

$$\begin{aligned} v \cos\theta= \left( \begin{matrix} v_{1}\cos\theta\\ v_{2} \cos\theta\\ v_{3} \cos\theta\end{matrix} \right)\#\left( 4 \right) \end{aligned}$$

$$\begin{aligned} \left( k\times v \right)\sin\theta=\left( \begin{matrix} \left( k_{2}v_{3}-k_{3}v_{2} \right)\sin\theta\\ \left( k_{3}v_{1}-k_{1}v_{3} \right)\sin\theta\\ \left( k_{1}v_{2}-k_{2}v_{1} \right)\sin\theta\end{matrix} \right)\#\left( 5 \right) \end{aligned}$$

$$\begin{aligned} k \left( k \cdot v \right) \left( 1 - \cos\theta\right) = \left( \begin{matrix} k_{1} \left( k_{1} v_{1} + k_{2} v_{2} + k_{3} v_{3} \right) \left( 1 - \cos\theta\right) \\ k_{2} \left( k_{1} v_{1} + k_{2} v_{2} + k_{3} v_{3} \right) \left( 1 - \cos\theta\right) \\ k_{3} \left( k_{1} v_{1} + k_{2} v_{2} + k_{3} v_{3} \right) \left( 1 - \cos\theta\right) \end{matrix} \right)\#\left( 6 \right) \end{aligned}$$

Substituting Equations (4), (5), and (6) into Equation (2) yields the following result:

$$v^{'}=\left( \begin{matrix} cos\theta+k_{1}^{2}\left( 1-cos\theta\right) & k_{1}k_{2}\left( 1-cos\theta\right)-k_{3}sin\theta& k_{1}k_{3}\left( 1-cos\theta\right)+k_{2}sin\theta\\ k_{2}k_{1}\left( 1-cos\theta\right)+k_{3}sin\theta& cos\theta+k_{2}^{2}\left( 1-cos\theta\right) & k_{2}k_{3}\left( 1-cos\theta\right)-k_{1}sin\theta\\ k_{3}k_{1}\left( 1-cos\theta\right)-k_{2}sin\theta& k_{3}k_{2}\left( 1-cos\theta\right)+k_{1}sin\theta& cos\theta+k_{3}^{2}\left( 1-cos\theta\right) \end{matrix} \right)\cdot\left( \begin{aligned} v_{1} \\ v_{2} \\ v_{3} \end{aligned} \right)$$

$$\begin{aligned} =R_{ro}\left( \theta\right)\left( \begin{aligned} v_{1} \\ v_{2} \\ v_{3} \end{aligned} \right)\#\left( 7 \right) \end{aligned}$$

From Equation (7), R_ro_(θ) can be derived as follows.

$$\begin{aligned} R_{ro}\left( \theta\right)=\left( 1-cos\theta\right)\left( \begin{matrix} k_{1}^{2} & k_{1}k_{2} & k_{1}k_{3} \\ k_{2}k_{1} & k_{2}^{2} & k_{2}k_{3} \\ k_{3}k_{1} & k_{3}k_{2} & k_{3}^{2} \end{matrix} \right)+\left( \begin{matrix} cos\theta& -k_{3}sin\theta& k_{2}sin\theta\\ k_{3}sin\theta& cos\theta& -k_{1}sin\theta\\ -k_{2}sin\theta& k_{1}sin\theta& cos\theta\end{matrix} \right)\#\left( 8 \right) \end{aligned}$$

The rotation matrices around the X, Y, and Z axes can be obtained by substituting k with (1,0,0), (0,1,0), and (0,0,1), respectively, in Equation (8). The rotation matrices for a rotation by θ degrees around each axis are as follows:

- Rotation around the X-axis:

$$\begin{aligned} R_{x}\left( \theta\right)=\left( \begin{matrix} 1 & 0 & 0 \\ 0 & cos\theta& -sin\theta\\ 0 & sin\theta& cos\theta\end{matrix} \right)\#\left( 9 \right) \end{aligned}$$

- Rotation around the Y-axis:

$$\begin{aligned} R_{y}\left( \theta\right)=\left( \begin{matrix} cos\theta& 0 & sin\theta\\ 0 & 1 & 0 \\ -sin\theta& 0 & cos\theta\end{matrix} \right)\#\left( 10 \right) \end{aligned}$$

- Rotation around the Z-axis:

$$\begin{aligned} R_{z}\left( \theta\right)=\left( \begin{matrix} cos\theta& -sin\theta& 0 \\ sin\theta& cos\theta& 0 \\ 0 & 0 & 1 \end{matrix} \right)\#\left( 11 \right) \end{aligned}$$

The combined rotation matrix resulting from successive rotations depends on the sequence of rotations. For a rotation sequence of α degrees around the X-axis, β degrees around the Y-axis, and γ degrees around the Z-axis, the resulting rotation matrix R_global_ is:

$$\begin{aligned} R_{global}=R_{z}\left( \gamma\right)R_{y}\left( \beta\right)R_{x}\left( \alpha\right)\#\left( 12 \right) \end{aligned}$$

In this case, the X-, Y-, and Z-axes remained fixed as the global axes throughout each rotation. Alternatively, when the X, Y, and Z axes rotate in tandem with each transformation, this approach is typically recognized as the Euler angle. Rotation starts with the global X-, Y-, and Z-axes; however, these axes rotate with the object. We refer to these as the local axes. When rotating by α degrees around the X-axis, followed by β degrees around the global Y-axis, and γ degrees around the global Z-axis, the rotation matrix (R_local_) is given by:

$$\begin{aligned} R_{local}=R_{x}\left( \alpha\right)R_{y}\left( \beta\right)R_{z}\left( \gamma\right)\#\left( 13 \right) \end{aligned}$$

When the X-, Y-, and Z-axes are treated as fixed, the order of multiplication in the rotation matrix is reversed. For a rotation of α, β, and γ degrees around the fixed X, Y, and Z axes, respectively, we have:

$$\begin{aligned} v^{'}=R_{global}v=R_{z}\left( \gamma\right)R_{y}\left( \beta\right)R_{x}\left( \alpha\right)v\#\left( 14 \right) \end{aligned}$$

Thus, if v and v′are known, it is possible to determine α, β, and γ by inverting this equation.

*1.2. Representation of object rotation*

In the global XYZ coordinate system, the rotation of an object's position can be expressed in terms of its unique XYZ coordinate axes (local coordinate axes). To define the intrinsic rotational axes of an object, we employed the following method: As described below, we initially plotted three points, A, B, and C, on the object. We defined the local X-axis as the vector from point C to point A (vector *CA*), the local Z-axis as the cross-product of vectors *CA* and *CB*, and the local Y-axis as the axis perpendicular to both the X- and Z-axes (Fig. 1). Provided that the positional relationship between these three points and the object is preserved, the rotation of the object can be described by the rotations around the local X-, Y-, and Z-axes.


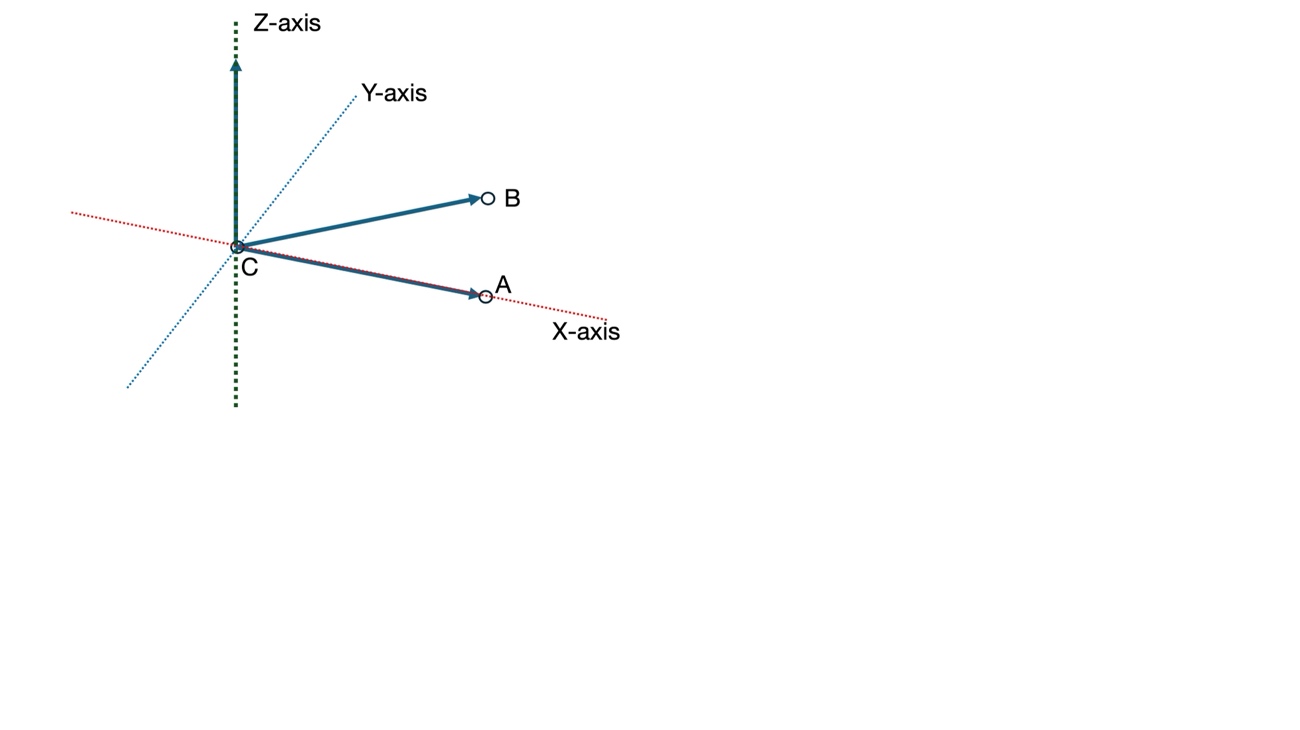
Fig. 1. Determination of the intrinsic coordinate axes of the model. After the arbitrary plotting of points A, B, and C on the model, the vector CA is defined as the Z-axis of the model, the axis perpendicular to both CA and CB is defined as its X-axis, and the axis perpendicular to both is defined as its Y-axis.

**2. Definition of the object coordinate system and creation of the rotation matrix**

Unique coordinate systems were defined for the bone models before and after osteotomy. These coordinate systems are specific to each model and distinct from the global coordinate system. Based on the coordinates obtained above, the XYZ coordinate system for the preosteotomy model can be expressed as follows:

$$\hat{X}= \frac{\vec{AO}}{\left| \vec{AO} \right|}$$

$$\hat{Z}=\frac{\vec{AO}\times\vec{BO}}{\left| \vec{AO}\times\vec{BO} \right|}$$

$$\begin{aligned} \hat{Y}=\hat{Z}\times\hat{X}\#\left( 15 \right) \end{aligned}$$

The post-osteotomy coordinate system X′Y′Z′ is similarly defined:

$$\hat{X'}= \frac{\vec{A'O}}{\left| \vec{A'O} \right|}$$

$$s\hat{Z'}=\frac{\vec{A'O}\times\vec{B'O}}{\left| \vec{A'O}\times\vec{B'O} \right|}$$

$$\begin{aligned} \hat{Y'}=\hat{Z'}\times\hat{X'}\#\left( 16 \right) \end{aligned}$$

The transformation from XYZ to X′Y′Z′ is defined as:

$$\begin{aligned} R=\left( \begin{matrix} \hat{X}\cdot\hat{X} & \hat{X}\cdot\hat{Y} & \hat{X}\cdot\hat{Z} \\ \hat{Y}\cdot\hat{X} & \hat{Y}\cdot\hat{Y} & \hat{Y}\cdot\hat{Z} \\ \hat{Z}\cdot\hat{X} & \hat{Z}\cdot\hat{Y} & \hat{Z}\cdot\hat{Z} \end{matrix} \right)\#\left( 17 \right) \end{aligned}$$

By substituting the values of points A, B, A’, and B’ from Section 2.3 into Equation (17), we can calculate the rotation matrix representing the osteotomy.

**Appendix B. Application to dome osteotomy, closed wedge osteotomy, and only coronal-plane correction**

**1. Application to 3D osteotomy: dome osteotomy as a model**

*1.1. Theory of dome osteotomy*

In dome osteotomy, the distal part to be corrected through rotation is cut hemispherically, allowing three degrees of rotational and translational freedom. Hemispherical osteotomy was performed approximately at the site of malunion, with rotation adjusted according to the calculated angles and shift aligned to maximize bone contact. Rotations were performed in the coronal, sagittal, and transverse planes (around the X-axis, Y-axis, and Z-axis, respectively). According to Equation (12), the rotation matrix R becomes.

$$\begin{aligned} R=R_{z}\left( \gamma\right)R_{y}\left( \beta\right)R_{x}\left( \alpha\right)=\left[ \begin{matrix} cos\gamma cos\beta& cos\gamma sin\beta sin\alpha-sin\gamma cos\alpha& cos\gamma sin\beta cos\alpha+sin\gamma sin\alpha\\ sin\gamma cos\beta& sin\gamma sin\beta sin\alpha+cos\gamma cos\alpha& sin\gamma sin\beta cos\alpha-cos\gamma sin\alpha\\ -sin\beta& cos\beta sin\alpha& cos\beta cos\alpha\end{matrix} \right]\#\left( 18 \right) \end{aligned}$$

Thus, from the factors of matrix R in Equation (17),

$$\begin{aligned} \left( \begin{matrix} \hat{X}\cdot\hat{X} & \hat{X}\cdot\hat{Y} & \hat{X}\cdot\hat{Z} \\ \hat{Y}\cdot\hat{X} & \hat{Y}\cdot\hat{Y} & \hat{Y}\cdot\hat{Z} \\ \hat{Z}\cdot\hat{X} & \hat{Z}\cdot\hat{Y} & \hat{Z}\cdot\hat{Z} \end{matrix} \right)=\left[ \begin{matrix} cos\gamma cos\beta& cos\gamma sin\beta sin\alpha-sin\gamma cos\alpha& cos\gamma sin\beta cos\alpha+sin\gamma sin\alpha\\ sin\gamma cos\beta& sin\gamma sin\beta sin\alpha+cos\gamma cos\alpha& sin\gamma sin\beta cos\alpha-cos\gamma sin\alpha\\ -sin\beta& cos\beta sin\alpha& cos\beta cos\alpha\end{matrix} \right]\#\left( 19 \right) \end{aligned}$$

is derived, and the rotation angles around each axis can be calculated using back substitution. The Python script for performing these calculations is provided in Appendix C.

*1.2. Surgical simulation (see Fig. 3 in the main text)*

Osteotomy was performed on a 10-year-old girl with a malunited supracondylar fracture of the humerus. The goal was to achieve anatomical alignment proximal to the unaffected side and plan a dome-shaped osteotomy in the supracondylar region. The coordinates of two arbitrary points on the model were A (−27.127, −61.196, −64.288), B (−38.499, −76.791), A’ (9.340, −16.915, −90.777), and B’ (−0.589, 16.862, −89.508). Substituting these into the script yielded rotation angles α = 33.93º, β = −23.01º, and γ = 9.06º. Based on the results, a dome osteotomy rotation simulation was performed using Rhino. Rotations were applied in the designated order, resulting in nearly perfect correction, except for the shift, which, when adjusted, confirmed that the correction was achieved as calculated.

**2. Application to 3D osteotomy: closed wedge osteotomy**

*2.1. Closed wedge osteotomy*

In current simulation techniques for 3D osteotomy, a closed-wedge osteotomy is performed by excising an area enclosed by a plane parallel to the transverse plane and another specific plane (Fig. 4 in the main text). Correction in the sagittal and coronal planes was achieved, and the osteotomy was completed by rotating the cut surfaces. For the upper limb, shortening up to 2 cm is acceptable, and in supracondylar humeral fractures, overgrowth due to the fracture allows closed-wedge osteotomy.

*2.2. Design of the closed wedge osteotomy*

The design and position of the osteotomy site are critical for this procedure. The osteotomy site was slightly proximal to the olecranon fossa. When viewed from the coronal plane, the distal side of the osteotomy plane is set perpendicular to the Z-axis, and the proximal one is rotated by α degrees around the X-axis to correspond to the deformity in the coronal plane. Next, based on the amount of deformity in the sagittal plane, the proximal osteotomy plane is tilted further by β degrees around the Y-axis to complete the proximal osteotomy plane. The area enclosed by these two osteotomy planes is then excised, and the distal bone fragment is rotated by γ degrees to correct rotational deformities.

*2.3. Calculation*

When correcting rotational deformities in the transverse plane, all rotation angles must be calculated in addition to corrections in the coronal and sagittal planes. In this case, coronal and sagittal rotations were performed using the global axes, and the rotation in the transverse plane uses the local Z-axis, which is altered by coronal and sagittal rotations. The rotations in the coronal and sagittal planes were calculated by multiplying Equations (9) and (10), and the rotation in the transverse plane was computed using Rodrigues’ rotation formula. Since the rotation in the coronal and sagittal planes is reproduced by Equation (11), the Z’ axis after these rotations is represented as:

$\begin{aligned} z^{'}=R_{y}\left( \beta\right)R_{x}\left( \alpha\right)z=\left( \begin{aligned} sin\beta cos\alpha\\ sin\alpha\\ cos\beta cos\alpha\end{aligned} \right)\#\left( 20 \right) \end{aligned}$

From the rotation matrix derived using Rodrigues’ formula (8), the total rotation matrix R_total_ representing all three plane rotations is:

$R_{total}=R_{ro}\left( \gamma\right)R_{y}\left( \beta\right)R_{x}\left( \alpha\right)$

$$\begin{aligned} =\left( \begin{matrix} cos\beta cos\gamma+sin\beta sin\alpha sin\gamma& -cos\beta sin\gamma+sin\beta sin\alpha cos\gamma& sin\beta cos\alpha\\ cos\alpha sin\gamma& cos\alpha cos\gamma& -sin\alpha\\ \cdot sin\beta cos\gamma+cos\beta sin\alpha sin\gamma& sin\beta sin\gamma+cos\beta sin\alpha cos\gamma& cos\beta cos\alpha\end{matrix} \right)\#\left( 21 \right) \end{aligned}$$

The required correction angle can be determined by back-calculating the vectors before and after correction. Equation (20) matches R_yxz_, yielding the same result as sequentially rotating around the Z-axis, X-axis, and Y-axis in sequence in the global coordinate system.

*2.4. Design and example of an application of wedge osteotomy planes*

The method described in Section 2.3 is applied in 3D virtual software to determine the osteotomy planes. In Rhino, the Gumball function allows the osteotomy plane to be rotated to the necessary position based on the required rotation amount of each axis. A proximal osteotomy plane was created based on the rotation values obtained from the script. The position of the osteotomy plane changed depending on the osteotomy site and rotation angle. The sizes of the excised bone fragments were measured for preoperative planning. The Python script is presented in Appendix A.

Wedge osteotomy was performed under the same conditions as in Section 3.2. The calculated values were α, β, and γ, each representing the respective angles. These values differ from those in the rotation matrix in Section 3.2. The osteotomy site was positioned proximal to the olecranon fossa and was set 15 mm from the medial epicondyle. The osteotomy volume was determined using CAD software. After performing the osteotomy and adjusting the shift, we confirmed that the planned correction was achieved.

**3. Limiting rotation planes: approximating the target with only X-axis rotation**

Flexion-extension deformities in the sagittal plane may be self-corrected in children aged <10 years. Thus, in some cases, only coronal-plane correction was performed, and the outcome was observed. In such situations, using a script based on three-plane deformities may underestimate the correction required in the coronal plane if the uncorrected planes are also affected. Therefore, we calculated the angle required for correction to approximate the post-correction vector as closely as possible using only one plane. This is formulated as a minimization problem where the triangle GAB is approximated to triangle G’A’B’ by adjusting the rotation angle θ around the X-axis. When the triangle obtained by applying the rotation θ around the X-axis to GAB is represented as GA’B, ’ we minimize:

$$f(\theta)=||R_{x}(\theta)\cdot A-C||^{2}+||R_{x}(\theta)\cdot B-D||^{2} \left( 22 \right)$$

Appendix B provides a numerical script that uses the Nelder–Mead (NM) method. In most cases, the degree of deformity (θ) does not differ significantly from the values (α) calculated in Appendix B.

**Appendix C. Practical implementation details**

**1. Creation of bone models**

CT scanning and segmentation were performed following the method previously used for the radius [8]. A CT scan (CANON MEDICAL SYSTEMS, Aquilion ONE 64 CT scanner, Otawara, Ibaraki, Japan, 80 kV, 100 mA) was performed with a slice thickness of 0.5 mm to scan both humeri. Then, bones were segmented using Mimics 21.0 (Materialise, Leuven, Belgium). The resulting 3D surface models were exported in STL (stereolithography) format and imported into Rhino 8.15 (Robert McNeel & Associates, Seattle, WA, USA) for both the unaffected and affected sides. The models were created in separate layers, and the unaffected bone was mirrored.

The axes of the model were defined on the mirrored unaffected bone using 3D-CAD software. The global z-axis represents the long axis of inertia of the humerus, with the transverse plane perpendicular to it. The line connecting the highest points of the medial and lateral epicondyles on the transverse plane defined the Y-axis, and the sagittal plane was perpendicular to it. The X-axis was perpendicular to both the Y- and Z-axes, with the coronal plane defined as perpendicular to the X-axis.

**2. Determining local coordinate axes of the humerus before and after correction by superimposing unaffected and affected models**

Centroid G of the unaffected 3D model was then calculated. Two arbitrary points, H and I, are plotted on an unaffected 3D model (Fig. 3 in the main text). Points H and I can be placed anywhere on the humerus, and we recommend that they be positioned distal to the malunion (e.g., at the highest points of the lateral and medial epicondyles) to minimize measurement errors. The 3D model of the affected side was superimposed, such that the proximal part near the malunion overlapped as closely as possible. The humeral head allows precise alignment because it is less influenced by the epiphyseal formation.

The four components, G, H, I, and the unaffected humerus 3D model (designated as Object O_1_), were then copied and moved while preserving all positional relationships. These components were aligned with the distal part of the affected humerus (the moved components are designated as Objects O_2_, which includes the copied 3D model of the unaffected humerus along with the moved points G,’ H,’ and I’). Aligning distal landmarks, such as the epiphyseal nucleus and medial epicondyle, enhances accuracy. In Rhino, fine adjustments in all three planes can be made using the Gumball tool, and software with best-fit functionality, such as Geomagic Studio (3D Systems, Rock Hill, SC, USA), facilitates alignment. Once aligned, Objects O_1_ and O_2_ are shifted so that the centroids G and G’ coincide at the origin. After moving the centroids to the origin, the coordinates of points G, H, G’, and H’ are recorded.

**3. Python scripts**

**3.1 Python script for dome and closed wedge osteotomies.**

Here is a Python script for calculating the rotation angles on each plane during dome osteotomy and closed wedge osteotomy. By inputting points H, I, H', and I' in lines 4–7 and executing the script, the rotation angles for each plane are calculated for the two osteotomies. H’ and I’ are represented as HH and II in the script.

To calculate the rotation, the local coordinate systems of the pre- and post-rotation bone models O_1_ and O_2​_ are determined. Using Equation (17), the rotation matrix representing the transformation is calculated. The values of α, β, and γ can then be computed using Equations (19) and (21). The script's functionality was verified using Google Collaboratory (Google Inc., Mountain View, CA).

| 1. import numpy as np 2. # Coordinates of points H, I, HH, II, and O, and compute vectors 3. H = np.array([_,_,_]) 4. I = np.array([_,_,_]) 5. HH = np.array([_,_,_]) 6. II = np.array([_,_,_]) 7. O = np.array([0.0, 0.0, 0.0]) 8. AO = H - O 9. BO = I - O 10. CO = HH - O 11. DO = II – O 12. # Normalize vectors to create a local coordinate system for the original 13. X = AO / np.linalg.norm(AO) 14. Z = np.cross(AO, BO) 15. Z = Z / np.linalg.norm(Z) 16. Y = np.cross(Z, X) 17. X_prime = CO / np.linalg.norm(CO) 18. Z_prime = np.cross(CO, DO) 19. Z_prime = Z_prime / np.linalg.norm(Z_prime) 20. Y_prime = np.cross(Z_prime, X_prime) 21. # Create a rotation matrix from the local coordinate system 22. R_original = np.column_stack((X, Y, Z)) 23. R_rotated = np.column_stack((X_prime, Y_prime, Z_prime)) 24. # Calculate the rotation matrix that rotates R_original to R_rotated 25. R = R_rotated @ R_original.T 26. # Extract angles from the rotation matrix R using the ZYX convention 27. output1 = np.zeros(3) 28. output1[0] = np.degrees(np.arctan(R[2, 1] / R[2, 2])) 29. output1[1] = np.degrees(np.arcsin(-R[2, 0])) 30. output1[2] = np.degrees(np.arctan(R[1, 0] / R[0, 0])) 31. # Extract angles from the rotation matrix R using the YXZ convention 32. output2 = np.zeros(3) 33. output2[0] = np.degrees(np.arcsin(-R[1, 2])) 34. output2[1] = np.degrees(np.arctan(R[0, 2] / R[2, 2])) 35. output2[2] = np.degrees(np.arctan(R[1, 0] / R[1, 1])) 36. # Output results 37. print ('Dome Osteotomy X Y Z degrees') 38. print (output1) 39. print ('Wedge Osteotomy X Y Z degrees') 40. print (output2) |
| --- |

**Output Example**

The following values are assigned to H, I, HH, and II.

| H = np.array([-27.127, -61.196, -64.288])  I = np.array([-38.499, -30.286, -76.791])  HH = np.array([9.340, -16.915, -90.777])  II = np.array([-0.589, 16.862, -89.508]) |
| --- |

In this case, the results output by the script are as follows:

| Dome Osteotomy X Y Z degrees  [ 33.22027641 -24.08928224 -5.33117274]  Wedge Osteotomy X Y Z degrees  [ 30.91488274 -27.10404247 -5.67389881] |
| --- |

**3.2 Python script for only coronal-plane correction.**

Here is a script for calculating the optimal rotation angle to achieve the desired alignment only in the coronal plane. Specifically, it determines the angle θ at which the triangle GHI should be rotated about the X-axis to approximate triangle GH'I′. Using Equation (22), the value of θ is computed by minimizing f(θ) with the Nelder–Mead method. By changing the method specified in method (line 34), it is possible to employ alternative numerical approaches.

| 1. import numpy as np 2. from scipy.optimize import minimize 3. # Coordinates of points H, I, HH, II, and O, and compute vectors 4. H = np.array([_,_,_]) 5. I = np.array([_,_,_]) 6. HH = np.array([_,_,_]) 7. II = np.array([_,_,_]) 8. O = np.array([0.0, 0.0, 0.0]) 9. # Define the rotation matrix around the X-axis 10. def rotation_x(theta): 11. cos_theta = np.cos(theta[0]) 12. sin_theta = np.sin(theta[0]) 13. return np.array([ 14. [1, 0, 0], 15. [0, cos_theta, -sin_theta], 16. [0, sin_theta, cos_theta] 17. ]) 18. # Define the objective function to minimize 19. def objective(theta): 20. # Rotate points H and I around the X-axis by angle theta 21. R_x = rotation_x(theta) 22. H_rotated = R_x @ H 23. I_rotated = R_x @ I 24. # Calculate the sum of squared distances between (H_rotated, HH) and (I_rotated, II) 25. distance_H = np.linalg.norm(H_rotated - HH) 26. distance_I = np.linalg.norm(I_rotated - II) 27. return distance_H **2 + distance_I**2 28. # Perform the optimization to find the angle that minimizes the objective function 29. result = minimize(objective, [0.0], method='Nelder-Mead') 30. # Output results] 31. print ('Optimal angle (X)') 32. print (np.degrees(result.x[0])) |
| --- |

**Output Example**

The following values are assigned to H, I, HH, and II.

| H = np.array([-27.127, -61.196, -64.288])  I = np.array([-38.499, -30.286, -76.791])  HH = np.array([9.340, -16.915, -90.777])  II = np.array([-0.589, 16.862, -89.508]) |
| --- |

In this case, the results output by the script are as follows:

| Optimal angle (X)  32.629946432700415 |
| --- |
